# Mechanisms Through Which Some Mitochondria-Generated Metabolites Act as Second Messengers That Are Essential Contributors to the Aging Process in Eukaryotes Across Phyla

**DOI:** 10.3389/fphys.2019.00461

**Published:** 2019-04-18

**Authors:** Paméla Dakik, Younes Medkour, Karamat Mohammad, Vladimir I. Titorenko

**Affiliations:** Department of Biology, Concordia University, Montreal, QC, Canada

**Keywords:** mitochondria, metabolism, signaling, interorganellar communications, hormetic stress response, redox homeostasis, aging, longevity

## Abstract

Recent studies have revealed that some low-molecular weight molecules produced in mitochondria are essential contributing factors to aging and aging-associated pathologies in evolutionarily distant eukaryotes. These molecules are intermediates or products of certain metabolic reactions that are activated in mitochondria in response to specific changes in the nutrient, stress, proliferation, or age status of the cell. After being released from mitochondria, these metabolites directly or indirectly change activities of a distinct set of protein sensors that reside in various cellular locations outside of mitochondria. Because these protein sensors control the efficiencies of some pro- or anti-aging cellular processes, such changes in their activities allow to create a pro- or anti-aging cellular pattern. Thus, mitochondria can function as signaling platforms that respond to certain changes in cell stress and physiology by remodeling their metabolism and releasing a specific set of metabolites known as “mitobolites.” These mitobolites then define the pace of cellular and organismal aging because they regulate some longevity-defining processes taking place outside of mitochondria. In this review, we discuss recent progress in understanding mechanisms underlying the ability of mitochondria to function as such signaling platforms in aging and aging-associated diseases.

## Introduction

Because mitochondria generate the bulk of cellular ATP, the functional state of these organelles is essential for physiology of all eukaryotic organisms (Nunnari and Suomalainen, 2012). The critical role of mitochondria in human physiology and health is underscored by the fact that mitochondrial dysfunction can lead to the development of many inborn mitochondriopathies and can contribute to such complex human diseases as type 2 diabetes, cardiovascular diseases, neurodegenerative diseases, and many forms of cancer (Lin and Beal, 2006; Fulda et al., 2010; Green et al., 2011; Szendroedi et al., 2011; Costa and Scorrano, 2012; Picard et al., 2016; Herst et al., 2017; Picca et al., 2018). Mitochondria can respond to changes in the nutrient, stress, proliferation or age status of the cell by altering the rates of catabolic and anabolic reactions yielding many different metabolites (Green et al., 2011; Nunnari and Suomalainen, 2012; López-Otín et al., 2013). All or most of these mitochondria-generated metabolites can serve as precursors, intermediates, or cofactors of various metabolic pathways that take place inside and outside of mitochondria and produce nucleotides, amino acids, and lipids (Chandel, 2015a). A body of recent evidence indicates that some of these mitochondria-generated metabolites can also function as second messengers whose concentrations regulate longevity-defining processes in various cellular locations, thereby contributing to cellular and organismal aging (Leonov and Titorenko, 2013; Chandel, 2014; Beach et al., 2015; Quirós et al., 2016; Matilainen et al., 2017). In common with second messengers of signal transduction (Gomperts et al., 2009; Cantley et al., 2014; Krauss, 2014; Lim et al., 2015), these mitochondria-produced second messengers of aging have the following properties: (1) they can be rapidly formed in response to certain extracellular signals, including various dietary, physiological, and pharmacological interventions or hormetic stresses; (2) they can be either the substrates of intracellular enzymatic reactions that are decelerated in response to these extracellular signals or the products of enzymatic reactions that are accelerated in response to such signals; (3) they can move within the cell from the place of their formation to a different cellular location or to several cellular locations; (4) they can be sensed by one or more ligand-specific protein(s) that can respond to the concentration changes of different second messengers by altering the rates and efficiencies of many cellular processes, thus amplifying and diversifying the second messenger signaling; and (5) they can be rapidly degraded (or inactivated in some other way) to allow a fast termination of the second messenger signaling (Leonov and Titorenko, 2013; Chandel, 2014; Beach et al., 2015; Quirós et al., 2016; Matilainen et al., 2017). One of the above properties of mitochondria-produced second messengers of aging, specifically their ability to undergo rapid degradation (or to be inactivated in a different manner), needs to be clarified for acetyl-CoA. Acetyl-CoA is a second messenger whose concentration outside mitochondria depends on both mitochondrial metabolite transport and the tricarboxylic acid (TCA) cycle in mitochondria (Eisenberg et al., 2014; Mariño et al., 2014; Schroeder et al., 2014; Peleg et al., 2016; Ye and Tu, 2018; see section “Some Mitochondrial TCA Cycle Intermediates Are Essential Contributors to the Aging Process” for more details). Acetyl-CoA is the only known donor of acetyl groups for a reversible posttranslational Nε-lysine acetylation of nuclear histones and many nonhistone proteins inside and outside of the nucleus; these nonhistone proteins have been implicated in cell cycle regulation, DNA damage repair, cellular signaling, protein folding, cytoskeleton organization, autophagy, and many other processes (Pietrocola et al., 2015a,b; Narita et al., 2019). Lysine acetyltransferases and lysine deacetylases (including the sirtuin family of NAD^+^-dependent deacetylases) enable a rapid posttranslational lysine acetylation and deacetylation (respectively) of both histones in the nucleus and nonhistone proteins inside and outside of the nucleus (Lapierre et al., 2015; van der Knaap and Verrijzer, 2016; Matilainen et al., 2017; Narita et al., 2019). Thus, lysine acetyltransferases and lysine deacetylases promote a fast termination of the acetyl-CoA-based second messenger signaling leading to protein acetylation. However, some epigenetic modifications of histones are known to be inherited in a transgenerational manner (Petruk et al., 2012; Greer et al., 2014; Kelly, 2014; Benayoun et al., 2015; Li and Casanueva, 2016). Although these heritable epigenetic modifications of histones have been revealed only for histone H3 methylation (which does not involve either acetyl-CoA or other mitochondria-generated metabolites) (Petruk et al., 2012; Greer et al., 2014; Kelly, 2014; Benayoun et al., 2015; Li and Casanueva, 2016) and acetylated histones are turned over within minutes (whereas methylated histones are turned over within days) (Jackson et al., 1975; Zee et al., 2010; Højfeldt et al., 2013; Ye and Tu, 2018), it is plausible that histone acetylation may be also inherited through generations. Thus, it is conceivable that the mitochondria-based acetyl-CoA signaling *via* histone acetylation may not always be rapidly inactivated to allow a fast termination of this kind of second messenger signaling.

The term “mitobolites” was coined for the metabolites of mitochondrial origin that operate as second-messenger signaling molecules in eukaryotes across phyla (Katewa et al., 2014). This essential role of mitochondria as signaling organelles in aging and aging-associated diseases has been conserved in the evolution of eukaryotes (Chandel, 2015b). Here, we discuss mechanisms through which mitochondria operate as such signaling organelles because they generate a distinct set of mitobolites that contribute to aging by regulating longevity-defining processes in cellular locations outside of mitochondria.

## Mitobolites that Contribute to Aging by Regulating Cellular Processes Outside of Mitochondria

### Mitochondria Control NAD^+^ Concentrations in Other Cellular Locations

**Figure 1:**
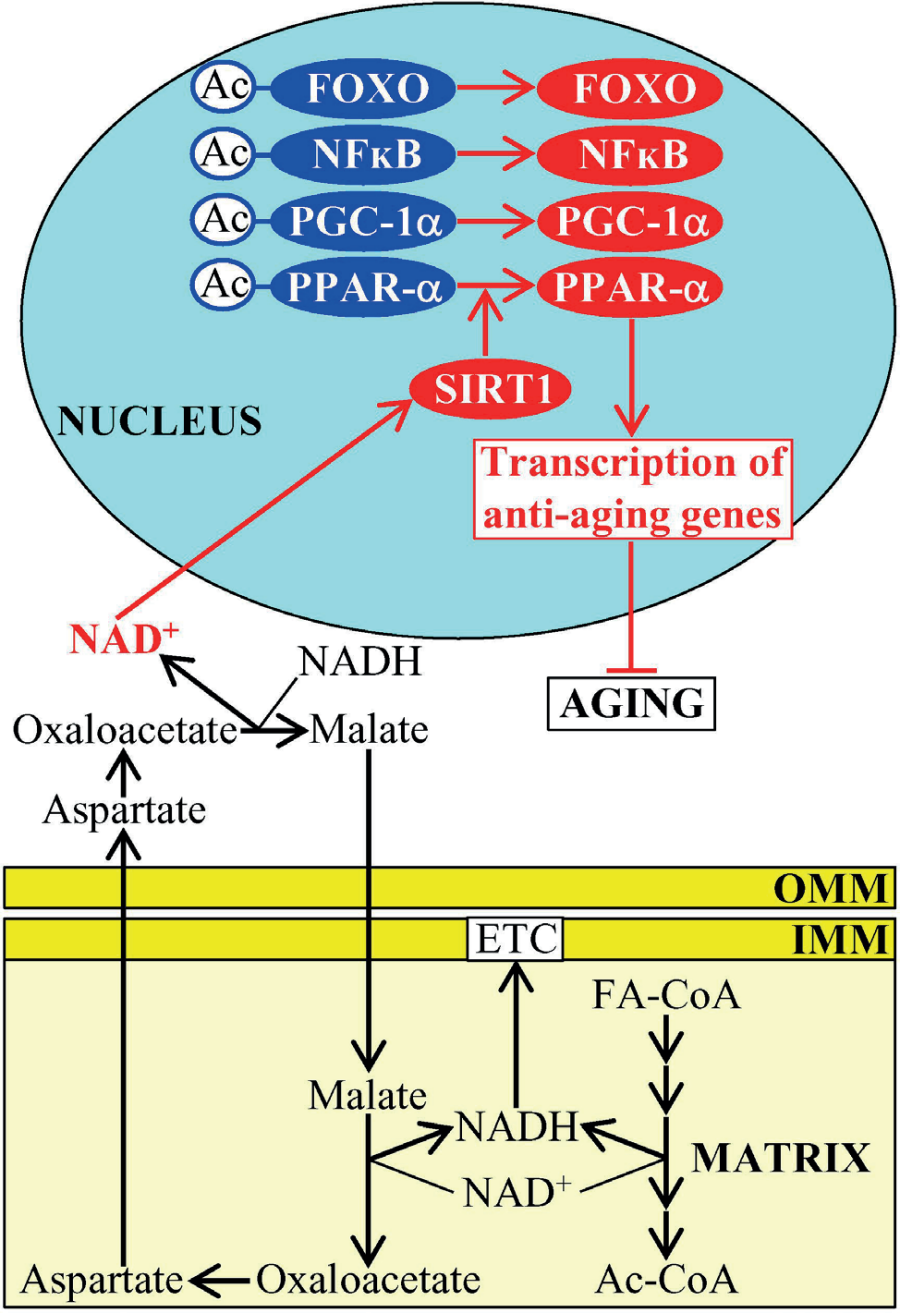
Mitochondrial metabolism influences NAD^+^ concentrations in other cellular locations, thus contributing to the aging process. An activation of fatty acid oxidation and a malate-aspartate shuttle in mitochondria elicits an increase in the concentration of cytosolic NAD^+^, which then stimulates the NAD^+^-dependent type III deacetylase SIRT1 in the nucleus. Once stimulated, SIRT1 deacetylates and activates several transcription factors that promote expression of many nuclear genes encoding aging-delaying proteins. Activation arrows and inhibition bars denote pro-aging (displayed in blue color) or anti-aging processes (displayed in red color). Pro-aging or anti-aging proteins are displayed in blue or red color, respectively. See the text for additional details. Abbreviations: Ac, acetyl group; Ac-CoA, acetyl CoA; FA-CoA, fatty acyl-CoA ester; ETC, electron transport chain; FOXO, forkhead transcription factor; IMM, inner mitochondrial membrane; NFκB, nuclear factor kappa B; OMM, outer mitochondrial membrane; PGC-1α, peroxisome proliferator-activated receptor gamma coactivator 1-alpha; PPAR-α, peroxisome proliferator-activated receptor alpha; SIRT1, sirtuin (silent mating type information regulation 2 homolog) type 1.

Aging-delaying dietary (fasting), physiological (exercise), and pharmacological (metformin) interventions that activate the AMP-activated protein kinase (AMPK) are known to stimulate both mitochondrial fatty acid oxidation and a malate-aspartate shuttle in the mitochondrial membranes in mouse muscle (Cantó et al., 2009). This causes a significant increase in the concentration of cytosolic NAD^+^ and a subsequent activation of the NAD^+^-dependent type III deacetylase SIRT1 in the nucleus (Figure 1; Cantó et al., 2009; Cantó and Auwerx, 2009; Libert and Guarente, 2013). The NAD^+^-activated SIRT1 then deacetylates and stimulates FOXO1, FOXO3a, NFκB, PGC-1α, and PPAR-α transcription factors, which next activate transcription of many nuclear genes whose protein products in mammals decelerate cellular aging and delay the age-related onset of type 2 diabetes and other aging-associated metabolic syndrome diseases (Figure 1; Cantó et al., 2009; Cantó and Auwerx, 2009; Libert and Guarente, 2013; Herzig and Shaw, 2018). Of note, all these transcription factors play essential roles in inflammaging, a mild and chronic type of inflammation that contributes to the pathogenesis of most aging-associated diseases (Youssef and Badr, 2004; Zandbergen and Plutzky, 2007; Handschin and Spiegelman, 2008; Fan et al., 2010; Peng, 2010; Hwang et al., 2011; Dinulovic et al., 2016; Liu et al., 2017; Franceschi et al., 2018; Rea et al., 2018). In the nematode *Caenorhabditis elegans*, the NAD^+^-activated SIRT1 stimulates the forkhead (FOXO) transcription factor DAF-16 to promote expression of many nuclear genes encoding aging-delaying proteins and activates the anti-aging mitochondrial unfolded protein response (UPR^mt^) pathway (Mouchiroud et al., 2013).

### Mitochondria-Generated NADPH Helps to Maintain Intracellular Redox Homeostasis

**Figure 2:**
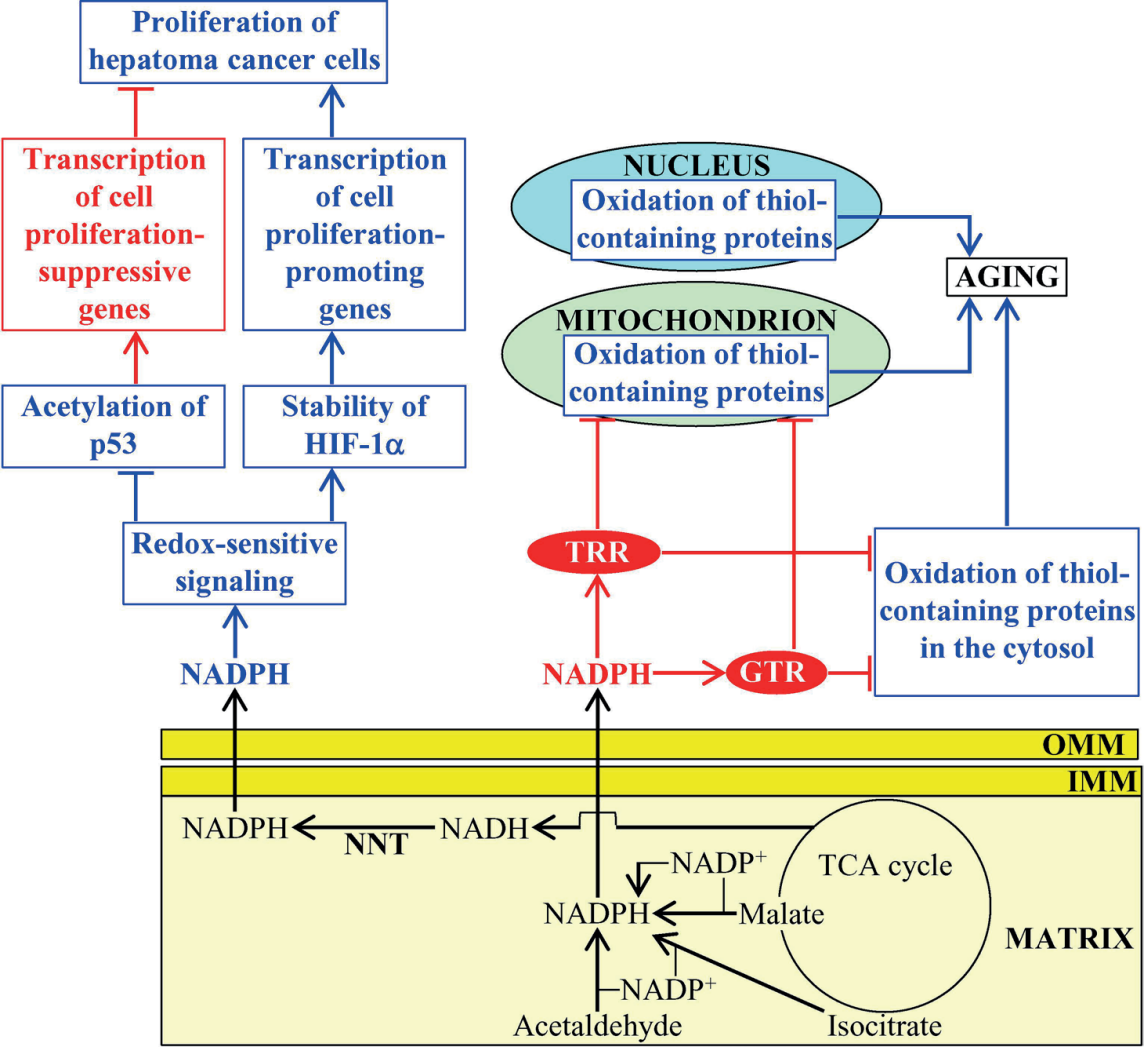
NADPH produced in mitochondria has a significant impact on aging and aging-associated diseases. In budding yeast, mitochondria-generated NADPH donates electrons for the thioredoxin and glutathione reductase systems (TRR and GTR, respectively), both of which delay aging by preventing oxidative damage of many thiol-containing proteins in mitochondria, the nucleus, and the cytosol. In mammals, the nicotinamide nucleotide transhydrogenase (NNT)-dependent production of mitochondrial NADPH contributes to tumor suppressor protein p53 acetylation, transcription factor HIF-1α (hypoxia-inducible factor 1α) stability, p53-dependent transcription of cell proliferation-suppressive genes, and HIF-1α-dependent transcription of cell proliferation-promoting genes in cancer cells. Activation arrows and inhibition bars for NADPH molecules that are shown in red color denote pro-aging (displayed in blue color) or anti-aging (displayed in red color) processes, and anti-aging proteins are displayed in red color. Activation arrows and inhibition bars for NADPH molecules that are shown in blue color denote pro-cancer (displayed in blue color) or anticancer (displayed in red color) processes. See the text for additional details. Abbreviations: IMM, inner mitochondrial membrane; OMM, outer mitochondrial membrane.

NADPH is generated in mitochondria of the yeast *Saccharomyces cerevisiae* in four different chemical reactions from the isocitrate and malate intermediates of the TCA cycle, as well as from acetaldehyde and NADH (Fraenkel, 2011; Cai and Tu, 2012). The vital metabolic function of this mitochondria-generated NADPH consists in supporting growth and viability of yeast by providing reducing equivalents for the synthesis of fatty acids, sterol lipids, and some amino acids (Fraenkel, 2011; Cai and Tu, 2012; Brandes et al., 2013). NADPH also acts as a second messenger that slows down yeast chronological aging (Brandes et al., 2013). This specific aging-decelerating role of NADPH is due to its ability to donate electrons for the thioredoxin and glutathione reductase systems called TRR and GTR (Grant, 2001); both TRR and GTR play essential roles in the delay of yeast chronological aging under caloric restriction (CR) conditions because they protect many thiol-containing proteins from oxidative damage in mitochondria, the nucleus, and the cytosol (Figure 2; Barral, 2013; Brandes et al., 2013).

In mitochondria of mammalian cells, NADPH is produced by the isocitrate dehydrogenase 2, formyltetrahydrofolate dehydrogenase 2 (ALDH1L2), methylenetetrahydrofolate reductase (MLHFD1L), malic enzyme 3, and nicotinamide nucleotide transhydrogenase (NNT) (Murphy, 2012; Yin et al., 2012a; Fernandez-Marcos and Nóbrega-Pereira, 2016; Zhang et al., 2017). By providing electrons for the mitochondrial TRR and GTR systems, this mitochondria-generated pool of NADPH helps to lower oxidative damage to thiol-containing proteins within mitochondria (Murphy, 2012; Yin et al., 2012a; Fernandez-Marcos and Nóbrega-Pereira, 2016).

NNT activity, which is responsible for the production of more than 50% of mitochondrial NADPH (Rydström, 2006), is an essential contributor to the protection of mammalian cells from the mitochondria-controlled mode of apoptotic death (Yin et al., 2012b). In mammals, NNT is also critical for the non-mitochondrial redox signaling that influences the maintenance of cellular redox homeostasis and modulates transcription of nuclear genes involved in the proliferation of cancer cells. In fact, the NNT-dependent production of mitochondrial NADPH is an important contributing factor to the extent of tumor suppressor protein p53 acetylation, transcription factor HIF-1α (hypoxia-inducible factor 1α) stability, p53-dependent transcription of cell proliferation-suppressive genes, and HIF-1α-dependent transcription of cell proliferation-promoting genes in hepatoma cancer cells (Figure 2; Ho et al., 2017). The NNT-dependent production of mitochondrial NADPH is also essential for supporting a high demand of melanoma and carcinoma cancer cells in glutamine-derived carbons needed to support the proliferation of these cells (Gameiro et al., 2013). Furthermore, the activities of two other enzymes involved in NADPH production within mitochondria, namely ALDH1L2 and MLHFD1L, are essential for the ability of melanoma cancer cells to metastasize (Piskounova et al., 2015). Thus, the formation of NADPH in mitochondria is an essential contributor to cell proliferation and metastatic tumor formation in several forms of cancer, an aging-associated disease affecting over 120 million people worldwide (GBD 2017 Disease and Injury Incidence and Prevalence Collaborators, 2018).

Moreover, in response to a pathologically excessive cardiac workload, NNT in mitochondria of mice reverses its conventional (i.e., NADPH-producing) forward mode (Nickel et al., 2015). Such reversal of NNT mode impairs antioxidant defenses, enhances oxidative damage, elicits cardiac remodeling, causes heart failure, and ultimately leads to death (Nickel et al., 2015). Importantly, a depletion of NNT protects mice from heart failure (which is an age-associated pathology that affects more than 25 million people worldwide; Kiyuna et al., 2018) and death (Nickel et al., 2015).

### Mitochondria Control AMP Concentrations Outside of Mitochondria

**Figure 3:**
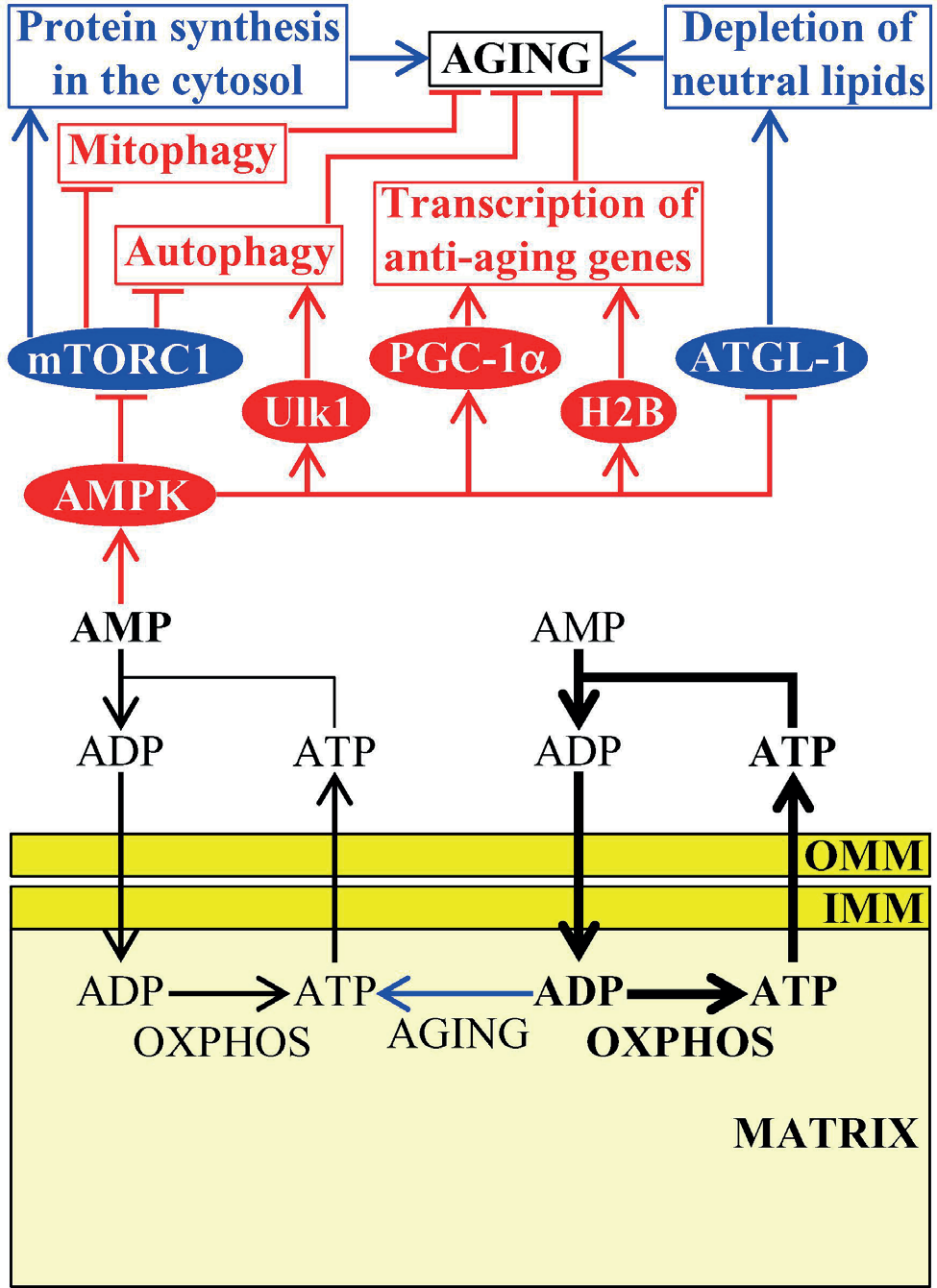
Mitochondria-dependent modulation of AMP concentration in the cytosol is involved in aging. An aging-associated decline in mitochondrial functionality causes a rise in the concentration of AMP outside of mitochondria. AMP then activates AMPK, which phosphorylates and changes activities of several proteins that have a significant impact on aging by influencing several longevity-defining cellular processes. Activation arrows and inhibition bars denote pro-aging (displayed in blue color) or anti-aging processes (displayed in red color). Pro-aging or anti-aging proteins are displayed in blue or red color, respectively. See the text for additional details. Abbreviations: AMPK, AMP-activated protein kinase; ATGL-1, adipose triglyceride lipase 1; H2B, histone 2B; IMM, inner mitochondrial membrane; mTORC1, mammalian (or mechanistic) target of rapamycin complex 1; OMM, outer mitochondrial membrane; OXPHOS, oxidative phosphorylation; PGC-1α, peroxisome proliferator-activated receptor gamma coactivator 1-alpha; ULK1, Unc-51 like autophagy activating kinase 1.

Due to an aging-associated decline in mitochondrial functionality, the concentration of mitochondria-produced cellular ATP also declines with age whereas the concentration of AMP outside of mitochondria rises; such aging-associated increase in cellular AMP stimulates protein kinase activity of AMPK in evolutionarily distant eukaryotes (Figure 3; Herzig and Shaw, 2018). As mentioned above, protein kinase activity of AMPK in mice can also be stimulated in response to certain aging-delaying dietary, physiological, and pharmacological interventions (Cantó et al., 2009; Garcia and Shaw, 2017). Once stimulated, AMPK decelerates cellular aging in mammals by phosphorylating the following downstream protein targets: (1) the mammalian (also known as mechanistic) target of rapamycin (mTOR) serine/threonine kinase, thus inhibiting the mTOR complex 1 (mTORC1); this suppresses the pro-aging process of protein synthesis in the cytosol (which is activated by mTORC1) and promotes the anti-aging processes of autophagy and mitophagy (both of which are downregulated by mTORC1) (Xu et al., 2012; Garcia and Shaw, 2017; Herzig and Shaw, 2018); (2) the autophagy-initiating protein kinase Ulk1, thus stimulating the anti-aging process of autophagy (Kim et al., 2011); (3) the transcription factor PGC-1α, thus promoting transcription of many nuclear genes that encode proteins involved in mitochondrial biogenesis and in other aging-delaying cellular processes (Garcia and Shaw, 2017; Herzig and Shaw, 2018); and (4) histone H2B, thus initiating the establishment of an aging-delaying transcriptional pattern that is essential for the survival of cells exposed to various metabolic and environmental stresses (Figure 3; Bungard et al., 2010). It is conceivable that the resulting stimulation of autophagy, mitophagy, and mitochondrial biogenesis may provide a compensatory mechanism for eliminating “old,” dysfunctional mitochondria and replacing them with “young,” functional ones. In the nematode *C. elegans*, stimulated AMPK phosphorylates and inhibits the adipose triglyceride lipase ATGL-1 in adipose-like tissues, thereby suppressing an age-related depletion of stored neutral lipids and delaying organismal aging (Figure 3; Narbonne and Roy, 2009; Xie and Roy, 2015).

### Some Mitochondrial TCA Cycle Intermediates Are Essential Contributors to the Aging Process

**Figure 4:**
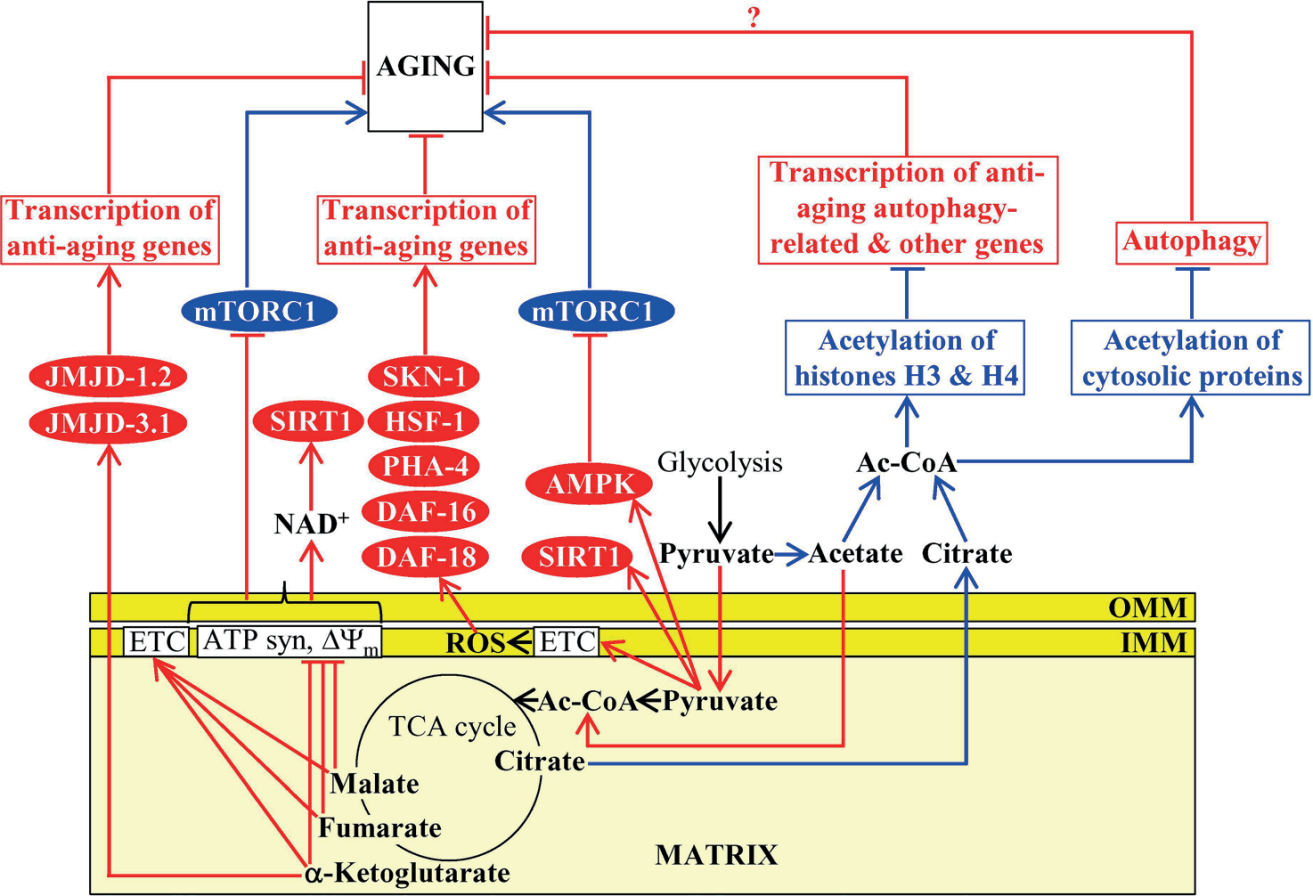
Mitochondria contribute to aging because they affect the homeostasis of mitochondrial TCA cycle intermediates. Some mitochondrial TCA cycle intermediates regulate activities of several non-mitochondrial proteins known for their essential roles in setting up an aging-delaying cellular pattern. Activation arrows and inhibition bars denote pro-aging (displayed in blue color) or anti-aging processes (displayed in red color). Pro-aging or anti-aging proteins are displayed in blue or red color, respectively. See the text for additional details. Abbreviations: Ac-CoA, acetyl CoA; AMPK, AMP-activated protein kinase; ATP syn, ATP synthase; DAF-16, forkhead box protein O; DAF-18, phosphatidylinositol 3,4,5-trisphosphate 3-phosphatase and dual-specificity protein phosphatase; ETC, electron transport chain; HSF-1, heat shock factor 1; IMM, inner mitochondrial membrane; JMJD-1.2 and JMJD-3.1, JmjC-domain-containing histone lysine demethylases 1.2 and 3.1; mTORC1, mammalian (or mechanistic) target of rapamycin complex 1; OMM, outer mitochondrial membrane; PHA-4, defective pharyngeal development protein 4; SIRT1, sirtuin (silent mating type information regulation 2 homolog) type 1; SKN-1, skinhead-1 transcription factor; ΔΨ_m_, the electrochemical potential across the inner mitochondrial membrane.

The homeostasis of some mitochondrial TCA cycle intermediates and related metabolites is an essential contributing factor to aging and longevity of *C. elegans*. A rise in the concentration of pyruvate, a product of glycolysis that is transported to mitochondria and then converted into acetyl-CoA to feed the TCA cycle, causes longevity extension in this nematode worm (Mouchiroud et al., 2011). Data on the epistatic effects of mutations eliminating some longevity assurance proteins suggested the following two mechanisms for the longevity extension by the rise of pyruvate in mitochondria: (1) the rise of pyruvate simulates AMPK/AAK-2 and the sirtuin SIRT1/SIR-2.1, both of which are known for their essential pro-longevity roles; the mechanism of such stimulation remains unknown; and (2) the rise of pyruvate intensifies mitochondrial respiration and ROS production, thereby triggering a hormetic oxidative stress response to stimulate the lipid phosphatase DAF-18 as well as the transcription factors PHA-4, HSF-1, DAF-16, and SKN-1, all of which are essential pro-longevity factors; the mechanism underlying such pyruvate-driven stimulation is also presently unknown (Figure 4; Mouchiroud et al., 2011). Of note, pyruvate concentration is significantly increased in the exometabolome excreted to the external environment by long-lived Mit mutants of *C. elegans* that are impaired in some protein components of the mitochondrial electron transport chain (ETC), but not in the exometabolomes of wild-type nematodes and short-lived Byby mutants of *C. elegans* impaired in other mitochondrial ETC components (Butler et al., 2010, 2013; Mishur et al., 2016). If added exogenously, pyruvate extends longevity of wild-type nematodes by stabilizing the hypoxia-inducible factor 1 (HIF-1), a transcriptional activator of longevity-assurance nuclear genes promoting survival during hypoxia (Figure 4; Mishur et al., 2016). Given that exogenous pyruvate also increases the stability and enhances the transcriptional activity of HIF-1 in cultured mouse fibroblasts, it is conceivable that such aging-delaying effect of pyruvate on HIF-1 has been conserved in the evolution of eukaryotes (Mishur et al., 2016). The mechanism through which pyruvate can stabilize HIF-1 to extend *C. elegans* longevity remains to be established.

An exposure of *C. elegans* to exogenous α-ketoglutarate increases the endogenous concentration of this TCA cycle intermediate in worms and extends longevity (Chin et al., 2014). This endogenous α-ketoglutarate binds to the subunit β of ATP synthase, whose ability to synthesize ATP is coupled to both the activity of mitochondrial ETC and the proton-motive force across the inner mitochondrial membrane; such binding causes a partial suppression of both mitochondrial respiration and ATP synthesis (Chin et al., 2014). The α-ketoglutarate-driven decline in ATP concentration inhibits protein kinase activity of mTOR *via* presently unknown mechanism (Chin et al., 2014). Because mTOR suppresses autophagy (Laplante and Sabatini, 2012), its indirect inhibition by α-ketoglutarate in *C. elegans* delays aging and extends longevity by stimulating the anti-aging process of autophagic degradation (Figure 4; Chin et al., 2014). Moreover, α-ketoglutarate is an essential coenzyme of lysine-specific histone demethylases that contain the Jumonji C (JmjC) demethylase domain (Teperino et al., 2010). The JmjC-domain-containing histone lysine demethylases jmjd-1.2/PHF8 and jmjd-3.1/JMJD3 of *C. elegans* respond to mild mitochondrial stress by activating transcription of many nuclear genes whose protein products are integrated into the anti-aging UPR^mt^ pathway and extending nematode longevity (Merkwirth et al., 2016). It has been suggested that these longevity-extending effects of jmjd-1.2/PHF8 and jmjd-3.1/JMJD3 in *C. elegans* may be caused by an increase of α-ketoglutarate concentration in mitochondria undergoing an age-related partial decline of their functionality (Figure 4; Merkwirth et al., 2016).

A treatment of *C. elegans* with malate or fumarate extends longevity, likely because it allows to alleviate an aging-associated decline in the concentrations of these two intermediates of the TCA cycle in mitochondria (Edwards et al., 2013). Both malate or fumarate increase the concentration of NAD^+^ and the NAD^+^/NADH ratio because they (1) stimulate mitochondrial respiration, decrease mitochondrial membrane potential, lower mitochondrial ATP production, and cause a mild uncoupling of mitochondrial oxidative phosphorylation; and (2) activate the glyoxylate shunt, malate dismutation, and fumarate reduction in mitochondria (Edwards et al., 2013). The glyoxylate shunt, malate dismutation, fumarate reduction, and the NAD^+^-activated sirtuin SIR-2.1 are all required for longevity extension by malate and fumarate (Edwards et al., 2013). It is therefore believed that mitochondrial metabolism of these two TCA cycle intermediates allows to prevent an age-related decline in NAD^+^ concentration, thus promoting the SIR-2.1-driven aging-delaying cellular processes outside of mitochondria (Figure 4; Edwards et al., 2013).

An exposure of *C. elegans* to exogenously added oxaloacetate, a TCA cycle intermediate, extends longevity; it is presently unknown if such exposure can increase the endogenous concentration of oxaloacetate in mitochondria of the nematode (Williams et al., 2009). Both the guardian of metabolism and energy homeostasis AMPK/AAK-2 and the FOXO transcription factor DAF-16, but not the sirtuin SIRT1/SIR-2.1, are required for the observed oxaloacetate-dependent longevity extension of *C. elegans* (Williams et al., 2009). The mechanism through which exogenously added oxaloacetate can increase nematode life span requires further investigation.

Mitochondria in budding yeast, fruit flies, mice and cultured human cells influence acetyl-CoA concentration outside of mitochondria (Eisenberg et al., 2014; Mariño et al., 2014; Schroeder et al., 2014; Ye and Tu, 2018). Acetyl-CoA is not only a molecule that enters the TCA cycle following pyruvate decarboxylation in mitochondria but also the only known donor of the acetyl groups for a reversible posttranslational Nε-lysine acetylation of proteins in the cytosol and the nucleus (Pietrocola et al., 2015a,b; Matilainen et al., 2017; Narita et al., 2019).

In budding yeast, non-mitochondrial acetyl-CoA is generated from acetate (one of the products of glucose fermentation in this yeast; Fraenkel, 2011) in a reaction catalyzed by acetyl-CoA synthetase 2 (Acs2); this enzyme is confined to the cytosol and the nucleus (Takahashi et al., 2006). The efficiencies with which acetate and its precursor pyruvate are transported from the cytosol to mitochondria can define acetyl-CoA concentration outside of mitochondria. This is because such transport removes from the cytosol both the substrate of Acs2 and the metabolic precursor needed for substrate formation (Eisenberg et al., 2014; Schroeder et al., 2014). If the efficiency of acetate and pyruvate transport to mitochondria is low, Acs2 converts acetate to acetyl-CoA in the cytosol and the nucleus (Eisenberg et al., 2014; Schroeder et al., 2014). The Acs2-driven synthesis and accumulation of acetyl-CoA in the nucleus elicit histone H3 hyperacetylation (Figure 4; Eisenberg et al., 2014; Schroeder et al., 2014). This, in turn, represses transcription of the autophagy-related genes ATG5, ATG7, ATG11, and ATG14, thus suppressing the anti-aging process of autophagy and shortening life span of chronologically aging yeast (Figure 4; Eisenberg et al., 2014; Schroeder et al., 2014).

In fruit flies and mammals, non-mitochondrial acetyl-CoA is formed from acetate in a reaction catalyzed by acetyl-CoA synthetase short-chain family member 2 (ACSS2) and from citrate in a reaction catalyzed by ATP-citrate lyase (ACLY); both these reactions occur in the cytosol (Eisenberg et al., 2014; Mariño et al., 2014; Schroeder et al., 2014; Peleg et al., 2016; Ye and Tu, 2018; Narita et al., 2019). Citrate (a substrate of ACLY) derives from the mitochondrial TCA cycle and must be exported from mitochondria to be converted to acetyl-CoA, whereas the pool of acetate (a substrate of ACSS2) in the cytosol of fruit flies and mammals is not influenced by mitochondria (Eisenberg et al., 2014; Mariño et al., 2014; Schroeder et al., 2014; Peleg et al., 2016; Ye and Tu, 2018; Narita et al., 2019).

An aging-associated activation of the mitochondrial oxidative phosphorylation (OXPHOS) system, mitochondrial citrate synthase, and cytosolic ACLY in fruit flies elicits a rise in the concentration of citrate in the cytosol, thus making this TCA cycle intermediate accessible to ACLY and ultimately increasing acetyl-CoA concentration in the cytosol (Peleg et al., 2016). Acetyl-CoA then diffuses from the cytosol into the nucleus through the nuclear pores (Wellen and Thompson, 2012; Narita et al., 2019). In the nucleus of fruit flies, acetyl-CoA is used as the substrate of the histone H4K12-specific acetyltransferase Chameau (Peleg et al., 2016). The Chameau-dependent histone H4 acetylation establishes a pro-aging gene transcription pattern in the nucleus, thereby shortening the life span of fruit flies (Figure 4; Peleg et al., 2016). Autophagy is one of the anti-aging processes suppressed by such histone H4 acetylation in fruit flies (Figure 4; Mariño et al., 2014).

In mice and cultured human cells, the use of various genetic and pharmacological interventions that increase or decrease acetyl-CoA concentration in the cytosol (including genetic interventions inactivating ACSS2 or ACLY) has revealed that cytosolic acetyl-CoA is used as the donor of the acetyl group for the acetylation of many cellular proteins (Mariño et al., 2014). Such protein acetylation is catalyzed by the cytosolic acetyltransferase EP300 (E1A-binding protein p300) and causes a suppression of the anti-aging process of autophagy (Figure 4; Mariño et al., 2014). The mechanisms through which the EP300-dependent protein acetylation suppresses autophagy, likely by stimulating mTORC1 signaling, require further investigation (Mariño et al., 2014; Schroeder et al., 2014; Pietrocola et al., 2015a,b). Moreover, although it is conceivable that the EP300-dependent protein acetylation and the ensuing suppression of autophagy may accelerate aging in mice and humans (Figure 4), it is presently unknown.

### Mitochondria-Produced Amino Acids Modulate Target of Rapamycin Signaling

**Figure 5:**
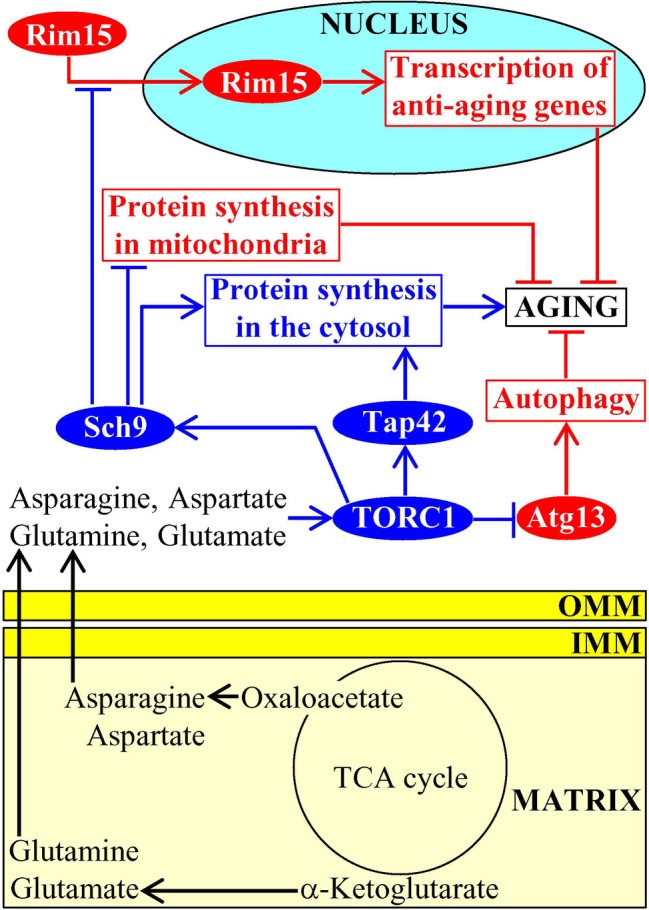
Amino acids synthesized in mitochondria accelerate aging by stimulating TOR signaling. After being synthesized in mitochondria and then exported to the cytosol, the amino acids aspartate, asparagine, glutamate, and glutamine stimulate protein kinase activity of TORC1 on the surface of vacuoles. Once activated, TORC1 initiates a development of an aging-accelerating cellular pattern by phosphorylating and altering activities of Sch9, Tap42, and Atg13. Activation arrows and inhibition bars denote pro-aging (displayed in blue color) or anti-aging processes (displayed in red color). Pro-aging or anti-aging proteins are displayed in blue or red color, respectively. See the text for additional details. Abbreviations: Atg13, autophagy-related protein 13; IMM, inner mitochondrial membrane; TORC1, target of rapamycin complex 1; OMM, outer mitochondrial membrane; Rim15, regulator of IME2 protein 15; Sch9, serine/threonine-protein kinase 9; Tap42, two A phosphatase associated protein 42.

Mitochondria of budding yeast house the synthesis of the amino acids aspartate, asparagine, glutamate, and glutamine from the TCA cycle intermediates oxaloacetate and α-ketoglutarate (Fraenkel, 2011; Cai and Tu, 2012; Chandel, 2015a). After being synthesized in mitochondria and then exported to the cytosol, these amino acids stimulate protein kinase activity of TORC1; this pro-aging protein complex in yeast cells resides on the surface of vacuoles (Figure 5; Crespo et al., 2002; Powers et al., 2006; Conrad et al., 2014; Swinnen et al., 2014). Once being stimulated by the amino acids generated in mitochondria, TORC1 phosphorylates and activates the downstream protein targets Sch9 and Tap42 (Swinnen et al., 2014; Eltschinger and Loewith, 2016). Sch9 and Tap42 then orchestrate a pro-aging cellular pattern in chronologically aging yeast because they control the following processes: (1) they both promote translation in the cytosol, a pro-aging process (Urban et al., 2007; Huber et al., 2009; Lee et al., 2009; Wei and Zheng, 2009; Broach, 2012); (2) Sch9 decelerates mitochondrial protein synthesis, an anti-aging process (Bonawitz et al., 2007; Pan and Shadel, 2009; Conrad et al., 2014; Swinnen et al., 2014); and (3) Sch9 suppresses nuclear import of Rim15, a nutrient-sensing protein kinase essential for the establishment of an anti-aging transcription program of nuclear genes (Figure 5; Roosen et al., 2005, Wanke et al., 2008; Smets et al., 2010). After being stimulated by the amino acids generated in mitochondria, TORC1 also phosphorylates Atg13; the TORC1-dependent phosphorylation of this autophagy-initiating protein inhibits the anti-aging process of autophagic degradation of dysfunctional organelles and macromolecules (Figure 5; Yorimitsu et al., 2007; Stephan et al., 2009, 2010; Laplante and Sabatini, 2012; Alers et al., 2014).

### Mitochondria-Generated Iron-Sulfur Clusters (ISC) Define Longevity of Replicatively Aging Yeast

ISC are synthesized and assembled in mitochondria of mammalian and yeast cells (Lill and Mühlenhoff, 2008; Xu and Møller, 2011; Lill et al., 2012; Paul and Lill, 2015). In mitochondria, ISC serve as essential cofactors of proteins involved in the TCA cycle and ETC as well as of proteins implicated in the synthesis of several amino acids, heme, molybdenum cofactor, lipoic acid, and biotin (Xu and Møller, 2011; Lill et al., 2012; Beilschmidt and Puccio, 2014; Stehling et al., 2014).

**Figure 6:**
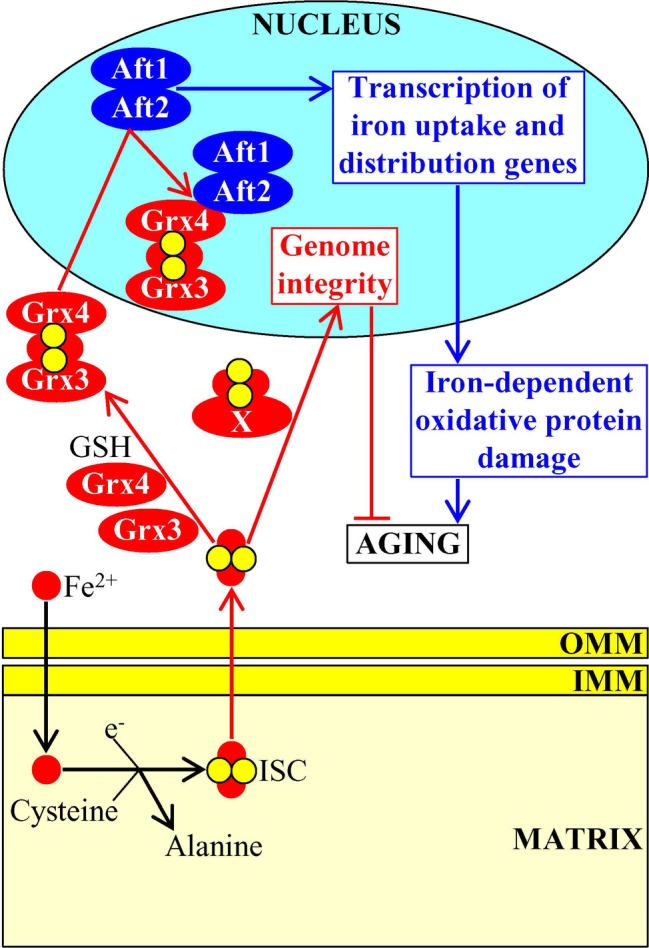
Mitochondria-generated iron-sulfur clusters (ISC) influence the aging process. Following their synthesis and assembly in mitochondria, ISC are exported to the cytosol where they bind to the monothiol glutaredoxins Grx3 and Grx4. This promotes the formation of Grx3/Grx4 dimers, stimulates nuclear import of Grx3/Grx4, suppresses transcription of genes involved in iron uptake and intracellular distribution, weakens the iron-dependent oxidative damage to proteins, and ultimately delays cellular aging. Some other ISC-containing proteins are also imported from the cytosol into the nucleus, where they support the maintenance of nuclear genome integrity. Activation arrows and inhibition bars denote pro-aging (displayed in blue color) or anti-aging processes (displayed in red color). Pro-aging or anti-aging proteins are displayed in blue or red color, respectively. See the text for additional details. Abbreviations: Aft1, activator of ferrous transport 1; Aft2, activator of ferrous transport 2; IMM, inner mitochondrial membrane; OMM, outer mitochondrial membrane.

ISC are also exported from the mitochondria to the cytosol where they become inorganic cofactors of many cytosolic and nuclear proteins (Xu and Møller, 2011; Lill et al., 2012; Paul and Lill, 2015). In the cytosol, ISC-containing proteins are essential components of such vital processes as amino acid synthesis, nucleotide metabolism, iron homeostasis regulation, xenobiotic metabolism, translation initiation, tRNA modification, and receptor tyrosine kinase signaling (Xu and Møller, 2011; Lill et al., 2012; Paul and Lill, 2015). The cytosolic pool of ISC defines the rate of yeast replicative aging because ISC insertion into the monothiol glutaredoxins Grx3 and Grx4 promotes the formation of Grx3/Grx4 dimers; Grx3/Grx4 are then imported into the nucleus, where these protein dimers bind to the transcription activators Aft1 and Aft2 of nuclear genes involved in iron uptake and intracellular distribution (Rutherford et al., 2005; Veatch et al., 2009; Ueta et al., 2012; Outten and Albetel, 2013). This binding causes dissociation of Aft1 and Aft2 from their target DNA, thereby decreasing the cellular concentration of free iron, weakening the iron-dependent oxidative damage to proteins and ultimately extending longevity of replicatively aging yeast (Figure 6; Veatch et al., 2009; Ueta et al., 2012; Outten and Albetel, 2013).

After acquiring ISC cofactors in the cytosol, some other ISC-containing proteins (perhaps, the nucleotide-excision repair helicase Rad3, the lagging-strand DNA synthesis primase Pri2 and/or the base-excision repair glycosylase Ntg2) are also imported into the nucleus (Veatch et al., 2009; Gottschling, 2012). These nuclear ISC-containing proteins define the rate of yeast replicative aging because they support the maintenance of nuclear genome integrity; this longevity assurance process relies on the involvement of the nuclear ISC proteins in the replication and repair of DNA and in the protection of telomeres (Figure 6; Veatch et al., 2009; Gottschling, 2012).

### Mitochondria Contribute to Aging-Associated Pathologies by Modulating Cytosolic Ca^2+^ Concentration

Mitochondria operate as signaling organelles in aging and aging-associated diseases also because they influence Ca^2+^ concentration in the cytosol. Although Ca^2+^ ions are known as potent and versatile second messengers in signaling pathways that orchestrate many cellular processes (Berridge et al., 2000; Clapham, 2007; Krebs, 2017), they are not metabolites and, therefore, not mitobolites. However, a body of evidence discussed in this section indicates that mitochondria are essential contributors to the maintenance of cellular Ca^2+^ homeostasis and that such contribution of mitochondria is linked to many aging-associated pathologies.

An uptake of extracellular Ca^2+^ by plasma membrane channels is the major external source of intracellular Ca^2+^ (Bagur and Hajnóczky, 2017; Calì et al., 2017; Krebs, 2017), whereas the endoplasmic reticulum (ER) (sarcoplasmic reticulum in muscle cells) and lysosomes are the two largest stores and major internal sources of Ca^2+^ within eukaryotic cells (Raffaello et al., 2016; Krebs, 2017; Giorgi et al., 2018). Because of the existence of the ER-mitochondria contact sites and due to the presence of Ca^2+^-selective channels in the mitochondrial membranes, mitochondria can transiently amass large amounts of Ca^2+^ ions within their matrix (Raffaello et al., 2016; Danese et al., 2017; Krebs, 2017; Muallem et al., 2017; Csordás et al., 2018; Giorgi et al., 2018; Pallafacchina et al., 2018; Paupe and Prudent, 2018). Instead of retaining these large amounts of Ca^2+^ ions for a long time, mitochondria use Ca^2+^ antiporters to release them into the cytosol in a highly regulated manner (Danese et al., 2017; Krebs, 2017; Giorgi et al., 2018; Pallafacchina et al., 2018). The coordinated uptake and efflux of Ca^2+^ by mitochondria are not only critical for the preservation of mitochondrial Ca^2+^ homeostasis but also important for the regulation of Ca^2+^ signaling within the cell, thus influencing cell life and death (Danese et al., 2017; Krebs, 2017; Giorgi et al., 2018; Pallafacchina et al., 2018; Verma et al., 2018). In fact, the impaired coordination between Ca^2+^ uptake by mitochondria *via* the ER-mitochondria contact sites and Ca^2+^ release by mitochondria into the cytosol has been implicated in many aging-associated pathologies, including obesity and insulin resistance, liver steatosis, Alzheimer disease, Parkinson disease, amyotrophic lateral sclerosis, breast cancer, colon and prostate cancer, hepatocellular carcinoma, and adrenocortical carcinoma (Raffaello et al., 2016; Danese et al., 2017; Krebs, 2017; Giorgi et al., 2018; Pallafacchina et al., 2018; Verma et al., 2018).

**Figure 7:**
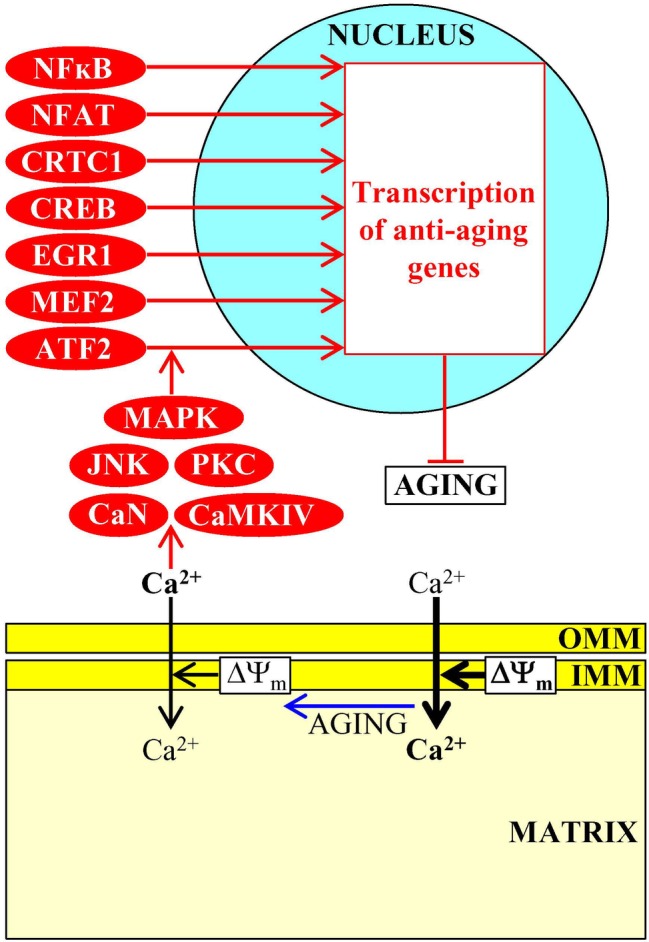
Ca^2+^ uptake and efflux by mitochondria contribute to aging-associated pathologies. An aging-associated decline in mitochondrial functionality causes a rise in cytosolic Ca^2+^ concentration. In the cytosol, Ca^2+^ initiates a cascade of events that establish an aging-delaying gene transcription pattern in the nucleus. Activation arrows and inhibition bars denote anti-aging processes and are displayed in red color. Anti-aging proteins are displayed in red color. See the text for additional details. Abbreviations: ATF2, activating transcription factor 2; CaMKIV, calmodulin-dependent protein kinase type IV; CaN, calcineurin; CREB, cAMP-responsive element binding protein; CRTC1, CREB-regulated transcription coactivator 1; EGR1, early growth response protein 1; IMM, inner mitochondrial membrane; JNK, c-Jun N-terminal protein kinase; MAPK, mitogen-activated protein kinase; MEF2, myocyte enhancer factor 2; NFAT, nuclear factor of activated T-cells; NFκB, nuclear factor kappa B; OMM, outer mitochondrial membrane; PKC, protein kinase C; ΔΨ_m_, the electrochemical potential across the inner mitochondrial membrane.

An aging-associated decline in mitochondrial functionality weakens the electrochemical potential across the inner mitochondrial membrane (ΔΨ_m_), thus decreasing a ΔΨ_m_- and uniporter-dependent import of cytosolic Ca^2+^ into mitochondria and rising cytosolic Ca^2+^ concentration in mammalian cells (Butow and Avadhani, 2004; Wojda et al., 2008). This, in turn, activates the Ca^2+^/calmodulin-dependent protein phosphatase calcineurin (CaN) and several Ca^2+^-dependent protein kinases (including protein kinase C [PKC], calmodulin-dependent protein kinase type IV [CaMKIV], c-Jun N-terminal protein kinase [JNK], and mitogen-activated protein kinase [MAPK]) (Butow and Avadhani, 2004; Wojda et al., 2008; Finley and Haigis, 2009). A combined action of these Ca^2+^-dependent protein phosphatase and protein kinases promotes nuclear import of the activating transcription factor 2 (ATF2), cAMP-responsive element binding protein (CREB) and its partner transcription coactivator protein 1 (CRTC1), myocyte enhancer factor 2 (MEF2), early growth response protein 1 (EGR1), nuclear factor of activated T-cells (NFAT), and nuclear factor kappa B (NFκB) (Figure 7; Butow and Avadhani, 2004; Wojda et al., 2008). The ensuing establishment of a specific transcription program driven by these transcription factors weakens the severity of some pathologies of old age, including aging-associated impairment of neuronal function, excitotoxicity and neurodegeneration, the immune imbalance and the cytokine dysregulation associated with inflammaging, hypertension, and hearing loss (Idrizbegovic et al., 2004; Toescu et al., 2004; Buchholz et al., 2007; Murchison and Griffith, 2007; Mellstrom et al., 2008; Celsi et al., 2009; Kidd Iii and Bao, 2012; Li et al., 2012; Behringer and Segal, 2017; Franceschi et al., 2018; Rea et al., 2018).

### The Dual Role of Mitochondria-Generated Reactive Oxygen Species (ROS) in Aging and Aging-Associated Diseases

ROS are mostly generated as superoxide by-products of several mitochondrial electron transport reactions, a hydrogen peroxide product of mitochondrial manganese superoxide dismutase, and a hydrogen peroxide product of cytochrome *c* oxidation by the mitochondrial intermembrane space (IMS) protein p66^shc^ (Giorgio et al., 2005, 2007; Brand, 2010). Mitochondria-generated ROS have a dual role in aging and aging-associated pathologies, as outlined below (Giorgio et al., 2007; Hekimi et al., 2011; Schieber and Chandel, 2014; Sun et al., 2016).

**Figure 8:**
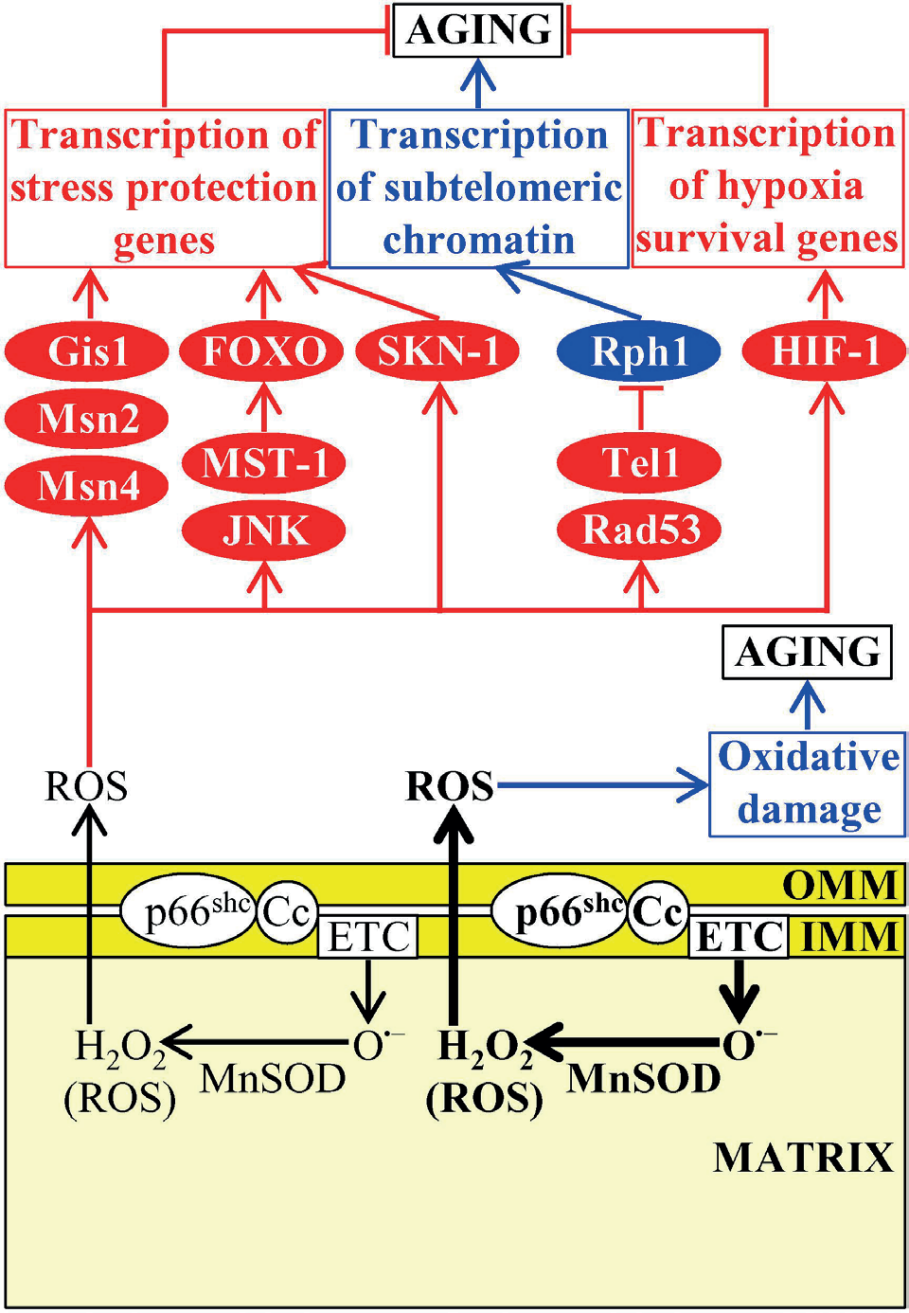
Mitochondria create and release reactive oxygen species (ROS), thereby affecting the aging process. Mitochondria-generated ROS are released into the cytosol and have a dual role in aging. If the concentration of mitochondria-derived ROS does not exceed a toxic threshold, ROS set up an aging-delaying cellular pattern by activating a “hormetic” signaling network that protects cells from an excessive oxidative stress and other age-related stresses. If the concentration of mitochondria-derived ROS exceeds a toxic threshold, excessive ROS quantities accelerate aging by directly damaging proteins, lipids, and nucleic acids inside and outside of mitochondria. Activation arrows and inhibition bars denote pro-aging (displayed in blue color) or anti-aging processes (displayed in red color). Pro-aging or anti-aging proteins are displayed in blue or red color, respectively. See the text for additional details. Abbreviations: Cc, cytochrome *c*; ETC, electron transport chain; FOXO, forkhead transcription factor; Gis1, GIg1-2 suppressor 1; IMM, inner mitochondrial membrane; JNK, c-Jun N-terminal protein kinase; MnSOD, manganese superoxide dismutase; Msn2, multicopy suppressor of SNF1 mutation protein 2; Msn4, multicopy suppressor of SNF1 mutation protein 4; MST-1, mammalian Ste20-like protein kinase 1; OMM, outer mitochondrial membrane; p66^shc^, SHC-transforming protein 1 having an additional N-terminal CH2 domain; Rad53, radiation sensitive protein 53; Rph1, regulator of PHR1; SKN-1, skinhead-1 transcription factor; Tel1, telomere maintenance protein 1.

If enzymatic and nonenzymatic antioxidant defense systems can maintain the concentration of mitochondria-derived ROS below a toxic threshold, ROS act as signaling molecules that initiate and sustain an aging-delaying cellular pattern by activating a “hormetic” signaling network (Giorgio et al., 2007; Hekimi et al., 2011; Longo et al., 2012; Ristow, 2014; Schieber and Chandel, 2014; Sun et al., 2016). This evolutionary conserved network protects cells from an excessive oxidative stress and from other age-related stresses and includes the following transcription factors, protein kinases, and metabolic enzymes: (1) the transcription factors Gis1, Msn2, and Msn4, which in chronologically aging budding yeast respond to sublethal ROS concentrations by activating expression of many nuclear genes involved in ROS decomposition, nutrient sensing, metabolism, stress protection, autophagy, mitochondrial ETC, cell cycle progression and transition to quiescence, and stationary phase survival (Causton et al., 2001; Fabrizio et al., 2001; Broach, 2012; De Virgilio, 2012; Schroeder and Shadel, 2014); (2) the DNA damage response protein kinases Tel1 and Rad53, whose successive action in response to hormetic ROS concentrations inactivates the Rph1-driven transcription of subtelomeric chromatin regions in the nucleus of chronologically aging budding yeast (Schroeder et al., 2013; Schroeder and Shadel, 2014; Shadel, 2014); (3) the mitochondrial base-excision repair enzyme Ntg1p, which in chronologically aging budding yeast responds to sublethal ROS concentrations by promoting mitochondrial genome protection from oxidative damage (Schroeder and Shadel, 2014); (4) the protein kinase JNK in nematodes and fruit flies, and the mammalian Ste20-like protein kinase 1 (MST-1) in nematodes; when activated in response to hormetic ROS concentrations, these protein kinases phosphorylate and stimulate the FOXO transcription activators of many nuclear genes implicated in oxidative stress and longevity assurance (Oh et al., 2005; Wang et al., 2005; Lehtinen et al., 2006; Greer and Brunet, 2008); (5) HIF-1 in nematodes and mammals, which in response to sublethal ROS concentrations activates transcription of longevity-assurance genes that promote survival during hypoxia (Bell et al., 2007; Zhang et al., 2009; Lee et al., 2010; Wang et al., 2010); (6) the transcription factor skinhead-1 (SKN-1) in nematodes, which responds to hormetic ROS concentrations by promoting transcription of genes essential for oxidative stress protection (Zarse et al., 2012); and (7) the enzymes Clk-1 and Mclk1, both of which are 5-demethoxyubiquinone hydroxylases involved in ubiquinone (coenzyme Q) synthesis in mitochondria of nematodes and mice, respectively; Clk-1 and Mclk1 extend longevity in response to hormetic ROS concentrations not because they are involved in ubiquinone biosynthesis but because they exhibit an additional activity in modulating the ETC, TCA cycle, ATP synthesis, and NAD^+^/NADH formation in mitochondria, thereby controlling the rates of ATP-dependent metabolic processes and NADH/NADPH-dependent oxidases that generate ROS in the cytosol, and ultimately defining the extent of ROS-dependent oxidative damage to cytosolic proteins (Figure 8; Felkai et al., 1999; Liu et al., 2005; Lapointe and Hekimi, 2008).

If enzymatic and nonenzymatic antioxidant defense systems are unable to sustain the concentration of mitochondria-derived ROS below a toxic threshold, excessive ROS quantities accelerate cellular and organismal aging by directly damaging proteins, lipids, and nucleic acids inside and outside of mitochondria (Figure 8; Giorgio et al., 2007; Hekimi et al., 2011; Schieber and Chandel, 2014). In replicatively aging budding yeast, a rise in mitochondria-generated ROS elicits the fragmentation and aggregation of mitochondria in the mother cells (Steinkraus et al., 2008; Longo et al., 2012; Denoth Lippuner et al., 2014). These fragmented and aggregated mitochondria fail to keep mitochondrial DNA, become dysfunctional, and are actively retained within the mother cells, thus limiting the replicative life span of these cells (McFaline-Figueroa et al., 2011; Hughes and Gottschling, 2012; Longo et al., 2012; Higuchi et al., 2013; Knorre et al., 2013; Denoth Lippuner et al., 2014; Nyström and Liu, 2014; Vevea et al., 2014).

## Conclusions

In this review, we analyzed mechanisms through which mitobolites, a distinct set of mitochondria-generated metabolites, can be released from mitochondria and then act as second messengers that contribute to cellular and organismal aging by regulating longevity-defining processes outside of mitochondria. Our analysis indicates that in eukaryotes across phyla, these second messengers of cellular aging exhibit the following common features: (1) they are produced in mitochondria in response to certain changes in the nutrient, stress, proliferation or age status of the cell; it remains unknown, however, what kind of protein sensors can respond to such changes by transmitting the signal to mitochondria so that mitochondria can increase the rates of catabolic and anabolic reactions producing these mitobolites; (2) their release from mitochondria and delivery to different non-mitochondrial locations within the cell also depend on these changes in the nutrient, stress, proliferation or age status of the cell; and (3) in cellular locations outside of mitochondria, they activate or inhibit a distinct set of protein sensors that in response set up a pro- or anti-aging cellular pattern by changing the efficiencies of certain pro- or anti-aging cellular processes; for some mitobolites, the identities of such protein sensors and/or mechanisms of their action are presently unknown. One important challenge is to define molecular mechanisms underlying the protein-mediated signaling cascades that trigger the production of certain mitobolites in response to specific changes in cell stress and physiology. The other challenge is to understand mechanisms that spatially and temporally integrate the formation of different mitobolites, their release from mitochondria and their subsequent action on various longevity-defining protein sensors outside of mitochondria. Future work will also aim at understanding how the essential contribution of mitobolites to aging is assimilated with other aspects of mitochondrial functionality that are not directly linked to the metabolism of low-molecular-weight molecules and that are known to contribute to the aging process through communications of mitochondria with various cellular compartments. These other aspects of mitochondrial functionality include the following: (1) a decline in ΔΨ_m_ and/or mitochondrial ETC, which in budding yeast, nematodes, fruit flies, and mammalian cells initiates the mitochondrial retrograde signaling pathway and/or decreases the TORC1 protein kinase activity; (2) the accumulation of unfolded or misfolded proteins within the IMS, which in nematodes and mammalian cells activates the UPR^mt^ pathway; (3) the formation of mitochondrial DNA (mtDNA) fragments in budding yeast and mice, which is followed by the migration of these fragments into the nucleus and by their insertion into nuclear DNA; (4) the processing, translation, and stabilization of mtDNA-encoded mRNAs in budding yeast; and (5) the tight coordination between mitochondria and the nucleus in transcribing genes, translating mRNAs, modifying mRNAs, and maintaining their stability, and selectively degrading certain proteins in budding yeast and mammalian cells (Caro et al., 2010; Leonov and Titorenko, 2013; Jazwinski, 2014; Beach et al., 2015; Martínez-Cisuelo et al., 2016; Quirós et al., 2016; Eisenberg-Bord and Schuldiner, 2017; Isaac et al., 2018).

## Author Contributions

PD, YM, KM, and VT wrote the text. VT prepared the figures.

### Conflict of Interest Statement

The authors declare that the research was conducted in the absence of any commercial or financial relationships that could be construed as a potential conflict of interest.
